# Transitional Locomotor Tasks in People With Mild to Moderate Parkinson's Disease

**DOI:** 10.3389/fneur.2020.00405

**Published:** 2020-05-15

**Authors:** Anna Kamieniarz, Justyna Michalska, Wojciech Marszałek, Anna Akbaş, Kajetan J. Słomka, Agnieszka Krzak-Kubica, Monika Rudzińska-Bar, Grzegorz Juras

**Affiliations:** ^1^Institute of Sport Sciences, Academy of Physical Education, Katowice, Poland; ^2^Department of Neurology, Medical University of Silesia in Katowice, University Clinical Center, Katowice, Poland; ^3^Department of Neurology, Faculty of Medicine and Health Sciences, Andrzej Frycz Modrzewski Kraków University, Kraków, Poland

**Keywords:** step task, gait initiation, Parkinson's disease, balance disorders, crossing obstacle

## Abstract

**Background:** People with Parkinson's disease (PD) exhibit deficits in maintaining balance both during quiet standing and during walking, turning, standing up from sitting, and step initiation.

**Objective:** The purpose of this study was to examine balance disorders during a transitional task under different conditions in participants with PD.

**Methods:** The research was conducted on 15 PD-II (mild) and 15 PD-III (moderate) individuals (H&Y II-III stage) and 30 healthy elderly. The transitional task was measured on two force platforms (A and B). The procedure consisted of three phases: (1) quiet standing on platform A, (2) crossing to platform B, and (3) quiet standing on platform B, each until measurements were completed. There were four conditions: crossing without an obstacle, crossing with an obstacle, and walking up and down the step.

**Results:** There were no significant differences between mild PD individuals and healthy elderly during quiet standing before the transitional task and after completing the task. The temporal aspects describing the different transitional tasks were comparable between mild PD and healthy subjects. Moderate PD participants presented a significantly higher COP velocity after the transitional task compared to the healthy older adults (*p* < 0.05). Additionally, the moderate PD group showed significantly higher values for transit time relative to healthy subjects during the transitional task in all conditions (*p* < 0.05).

**Conclusions:** Disease severity affects the temporal aspects of different transitional tasks in people with PD. The procedure of completing a transitional task under different conditions allowed differences between moderate and mild PD stages and healthy subjects to be observed.

## Introduction

Alterations in postural control strategies in Parkinson's disease have been typically investigated during standing tasks (1–4). However, there is clear evidence that balance disturbance may occur during dynamic tasks such as walking, turning, stair negotiation, and standing up from a chair (5–11). During the activities of daily living, disease-related balance disorders increase the risk of falls that may lead to injuries (12–14) and decrease the quality of life (QOL) among people with PD (15, 16).

Postural instability during level-ground walking, obstacle crossing, and ascending and descending stairs is widely described in the literature (6, 7, 17–21). There is evidence that people with PD present impaired postural control during gait with an additional task (17, 22, 23); therefore, a complex motor activity from daily life, such as crossing an obstacle or negotiating stairs, may be hazardous for people with PD. Moreover, it is known that individuals with PD commonly exhibit deficits in balance maintenance during transitions between states of static and dynamic balance (24, 25) which are associated with gait initiation (GI). There is evidence that both the anticipatory postural adjustment (APA) and the stepping phase of GI are impaired in PD (26). This is mainly due to akinesia, reported as slowness and poverty of movement (27). These functional limitations might increase the risk of falls during more challenging daily activities as a consequence of impaired transition from the double- to single-support phase (28).

Several studies have shown that people with PD have disturbances in APA prior to gait initiation (21, 26, 29–31), which is reduced and longer in duration with prolonged delays between APA onset and step onset (11, 32). While there is evidence that APA and postural control are impaired in PD before step initiation, still little is known about postural instability during a transitional task during different daily activities in PD. Only two reports have focused on gait initiation in PD before climbing stairs (21, 33), but not before obstacle crossing and descending stairs. It is known that the obstacle avoidance challenges control of the center of mass (COM), resulting in a greater COM motion in the anterior-posterior direction, a greater and faster COM motion in the vertical direction, and a greater distance between the COM and center of foot pressure (COP) (34). However, a previous study noted that participants with PD crossed the obstacle with their COM closer to their COP and a reduced forward COM movement (35). Hence, we assume that obstacle avoidance can impair the gait initiation by affects the anticipatory postural adjustments. Furthermore, there is evidence that postural instability and gait initiation impairments increase with PD severity (11). Studies have shown that more affected PD individuals (i.e., those with an H&Y score greater than 2.5) appear to limit their stability during gait initiation (11). Nevertheless, some authors suggest that the analysis of the transitional phase of a locomotor task might provide parameters useful for the characterization of early stage PD individuals (8). It is known that different stages of PD according to the H&Y scale present different motor impairments (36), thus there is a need to investigated them discretely. Although there are several reports separately investigating different stages of PD during a transitional task (8, 11), there is only one study comparing individuals with mild and moderate PD during obstacle negotiation (37). Therefore, an analysis of a transitional task under various conditions in patients with mild and moderate PD will allow for further exploration of the characteristics of this group. The resultant characteristics of the transitional task may provide useful information for clinicians to implement specific gait and balance exercises into rehabilitation programs for PD participants in each stage of the disease, as well as for monitoring the effects of interventions (32) aimed at decreasing the risk of falls and maintaining quality of life.

The aim of our study was to investigate balance disorders in people with mild and moderate PD during a transitional task under different conditions. We compared different aspects of transitional task between mild and moderate PD and healthy older adults. We hypothesized that balance disorders during the transitional phase would already be apparent in the mild PD stage with respect to healthy older adults. We also assumed that the temporal aspects of the applied transitional tasks would be able to differentiate between mild and moderate stages of PD.

## Materials and Methods

A case-control study was designed to investigate differences in a transitional task in participants with mild and moderate PD in comparison to healthy aged-matched controls. The participants were recruited from the Department of Neurology University Clinical Center Medical University of Silesia in Katowice, Poland. The study was approved by the ethics committee of the Academy of Physical Education in Katowice, Poland (No.7/2013/26.06.2013).

### Subjects

The experimental groups consisted of 30 subjects with idiopathic PD and 30 aged-matched healthy control subjects. The demographic and clinical characteristics of the subjects are presented in Table 1. The PD groups were classified into mild and moderate stages according to the Hoehn and Yahr Scale (H&Y) (36). On the basis of disease severity (II-III H&Y stages), PD participants were distributed into two groups: group PD-II, individuals at a mild clinical stage, and group PD-III, individuals at a moderate clinical stage. The PD-II and PD-III groups were different with respect to age, so the control subjects were divided on the basis of age into two groups: control group CA, healthy subjects age matched to group PD-II, and control group CB, healthy subjects age matched to group PD-III. All groups of participants provided a written informed consent before participating in the study. PD participants were tested during the “ON period” at their usual antiparkinsonian medication (at least one hour). At the time of testing, none of the participants exhibited any dyskinesia, dystonia, or other signs of involuntary movement, e.g., uncontrolled tics or short-lived, excessive movements such as a chorea. PD subjects were qualified for the study based on a clinical and psychological assessment carried out by a neurologist and psychologist from the Department of Neurology, University Clinical Center Medical University of Silesia in Katowice, Poland. The neurologist assessed PD severity using the Unified Parkinson's Disease Rating Scale (UPDRS) III (38). Additionally, the clinical assessment included H&Y stage classification and anamnesis about the history of neuromuscular or orthopedic impairments or neurological conditions other than PD that could affect balance and gait. Moreover, the psychologist assessed mental and cognitive impairments using the Mini-Mental State Examination (MMSE) (39).

**Table 1:** Demographic and clinical characteristics of the subjects.

	**Mild PD (PD-II)**	**Control group A (CA)**
*N*	15	15
Age [years]	61.9 ± 8.6	63.5 ± 4.3
Body mass [kg]	75.7 ± 9.0	70.2 ± 7.9
Body height [cm]	170.4 ± 6.7	167.3 ± 4.3
UPDRS - III [pts]	10.5 ± 3.4	-
H&Y stage	II	-
MMSE [pts]	28.1 ± 1.6	29.0 ± 1.6
	**Moderate PD (PD-III)**	**Control group B (CB)**
*N*	15	15
Age [years]	70.7 ± 4.5	71.5 ± 4.1
Body mass [kg]	75.1 ± 15.0	68.1 ± 9.8
Body height [cm]	167.7 ± 6.7	162.7 ± 10.3
UPDRS - III [pts]	28.9 ± 5.4	-
H&Y stage	III	-
MMSE [pts]	27.5 ± 2.5	28.2 ± 1.8

*UPDRS, Unified Parkinson's Disease Rating Scale; H&Y, Hoehn and Yahr Scale; MMSE, Mini-Mental State Examination*.

Inclusion criteria for PD participants consisted of (1) a diagnosis of PD, (2) a grading of stage II-III according to H&Y, and (3) being on antiparkinsonian drug treatment. The inclusion criteria for healthy subjects were (1) age over 50 years, (2) no history of neurodegenerative and other neurological impairments, and (3) consent to participate in the research. Exclusion criteria for both groups were (1) no consent to participate in the research, (2) dementia and cognitive impairment based on the results of the Mini-Mental State Examination (scores below 24), and (3) neuromuscular, vestibular, or orthopedic disorders.

### Transitional Task Procedure

**Figure 1:**
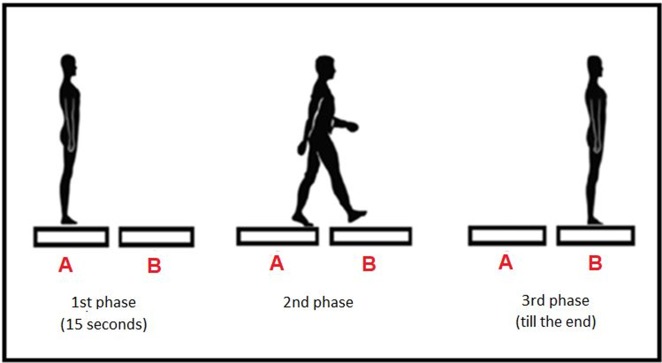
Testing procedure—three phases.

The transitional task was measured by two force platforms (AMTI, AccuGait). The platform sampling frequency was 100 Hz. The raw data was processed offline with a dual-pass 7 Hz low-pass Butterworth filter using MATLAB software (Mathworks, Natick, MA). The force platforms were marked as A and B, and were placed sagittally in a straight line in front of each other, and the distance between the force plates was 4 cm (Figure 1). This distance was dictated by the thickness of the obstacle used in the following trials in order to maintain similar step conditions throughout the trials.

The starting position in all trials was quiet standing with feet together, arms along the body, and eyes looking at a fixation point located on the wall.

**Figure 2:**
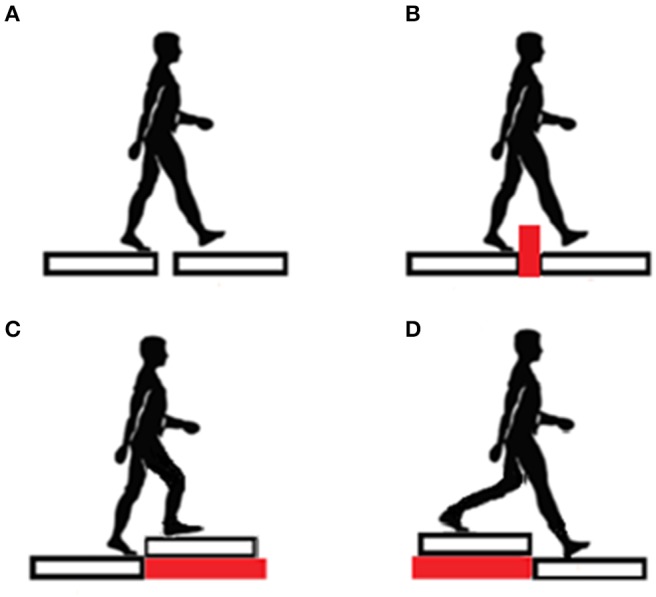
Four different conditions: **(A)** unperturbed crossing, **(B)** obstacle crossing, **(C)** step-up, **(D)** step-down.

The assessment of the transitional task comprised four different conditions (Figure 2) (40):

1. Trial 1 (unperturbed crossing): quiet standing on platform A for 15 s, one step transit to platform B (after an acoustic signal), followed by quiet standing until measurement completion.
2. Trial 2 (obstacle crossing): quiet standing on platform A for 15 s, then on a sound signal transit over the obstacle to platform B (one step), followed by quiet standing until measurement completion. A 16-cm high and 4-cm thick obstacle was inserted between the platforms.
3. Trial 3 (step-up): quiet standing on platform A for 15 s, then on a sound signal transit to platform B (one step up), followed by quiet standing until measurement completion. Platform B was placed on a 17-cm base directly at the edge of platform A.
4. Trial 4 (step-down): quiet standing on platform A for 15 s, then on a sound signal transit to platform B (one step down), followed by quiet standing until measurement completion. Platform A was placed on a 17-cm base directly at the edge of platform B.

The obstacle height and positioning height of platforms A and B were established based on the standard height of a curb (6–16 cm) and a step (15–17 cm) according to Polish building regulations.

Each trial lasted 35 s and was repeated three times, which was enough to achieve excellent reliability in all examined measures (ICC = 0.83–0.96) (41). The trial order was randomized. Before each trial, the subjects had time to practice stepping on the force platforms. The participants were instructed to start with the dominant leg.

The center of foot pressure (COP) displacement data was collected in three phases: phase 1–quiet standing before the transitional phase, phase 2–transitional phase, phase 3–quiet standing after making a step. The recording was divided into phases using an algorithm whose major components were foot contact with the platform and the limit of instantaneous COP displacement; beyond that point, exit from stability or stability gain were observed. The transitional phase started when a momentary COP displacement exceeded the mean COP displacement plus three SDs (the mean and SD were calculated based on measurements made within the first 5 s of the Phase 1). The step was over (stability regained) when the momentary COP displacement was lower than the mean value plus three SDs based on the last 5 s of the trial (Phase 3).

**Figure 3:**
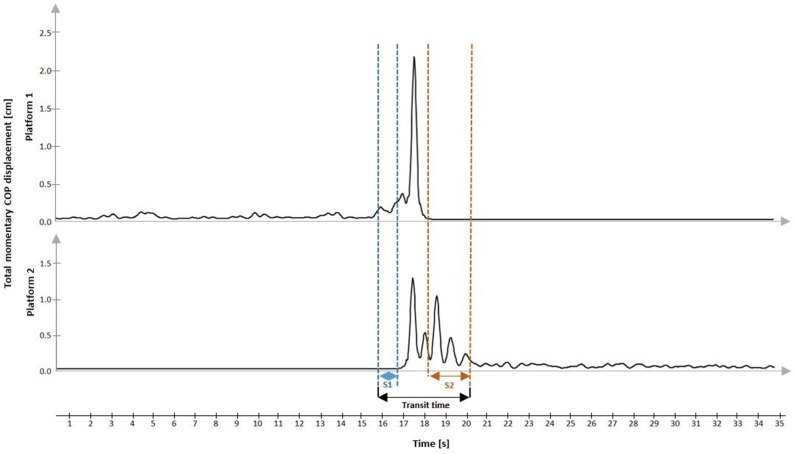
Representative COP displacement during the transitional trial.

The following variables were analyzed:

1. Phase 1 and Phase 3–quiet standing before and after transitional phase:
  – vCOP–the antero-posterior (AP) and medio-lateral (ML) COP velocity (cm/s)
2. Phase 2–transitional phase (Figure 3):
  – S1–the time from exiting steady standing to stepping on the second platform (s) (preparatory stability time); S1 includes the first step duration and APA period
  – S2–time from raising the foot from the first platform and contact on the second platform, until the stability was regained (s) (regained stability time)
  – transit time: time from exit from the stability state until gaining post-transit stability (s)

Transit time = the sum of S1 (s) + double-support period (s) + S2 (s). The double-support period is when each foot is in contact with one of the platforms (s).

### Statistical Analysis

The Shapiro-Wilk test was used to check the data for normal distribution. Variance homogeneity was assessed with Levene's test. The two-way ANOVA (group × testing condition) was used. Repeated measures one-way ANOVA was used to compare each group under particular testing conditions. *Post hoc* comparisons were performed using the Bonferroni test. The level of statistical significance was set at *p* ≤ 0.05 for all tests. Analyses were performed with Statistica, version 13.1.

## Results

The two-way interactions group × testing conditions were not significant for any of the study variables during phase 1, 2, and 3 (*p* > 0.05). The two-way ANOVA revealed a significant main group effect on phase 1 COP velocity for both sagittal plane [F_(3, 224)_ = 4.27; *p* = 0.006] and frontal plane [F_(3, 224)_ = 6.17; *p* < 0.001]. There was a significant main group effect on phase 3 COP velocity for both anterior-posterior direction [F_(3, 224)_ = 31.2; *p* < 0.001] and medio-lateral direction [F_(3, 224)_ = 19.06; *p* < 0.001]. Also, the two-way ANOVA revealed a significant main group effect on all of the variables in phase 2 (*p* < 0.05).

### Participants With Mild Parkinson's Disease

The phase 1 COP velocity for both the antero-posterior and medio-lateral directions did not differ between mild PD participants (PD-II) and healthy older people (CA) in all testing conditions (*p* > 0.05). However, there was a tendency toward lower COP velocity in both directions in mild PD. In PD-II, the transitional phase variables (transit time, S1, S2) were comparable between PD-II and CA in all conditions. The phase 3 COP velocity for both the antero-posterior and medio-lateral directions did not differ between mild PD participants and healthy older people (CA) for all testing conditions (Table 2).

**Table 2:** Intergroup comparison—PD-II vs. CA, PD-III vs. CB, and PD-II vs. PD-III.

			**Mild PD (PD-II)**	**Healthy subjects (CA)**	**Moderate PD (PD-III)**	**Healthy subjects (CB)**	**Intergroup comparison**			
**Variables**	**Trial phase**	**Trials**	**Mean (SD)**	**Mean (SD)**	**Mean (SD)**	**Mean (SD)**	**PD-II CA**	**PD-III vs. CB**	**PD-II vs. PD-III**	**CA vs. CB**
vCOP AP (cm/s)	Phase 1	Unperturbed	0.78 (0.28)	0.98 (0.41)	1.08 (0.44)	0.83 (0.17)	−	−	+	−
		Obstacle	0.82 (0.30)	0.95 (0.30)	1.06 (0.34)	1.08 (0,32)	−	−	−	−
		Step−up	0.95 (0.47)	0.96 (0.24)	1.03 (0.38)	1.11 (0.32)	−	−	−	−
		Step−down	1.01 (0.32)	1.10 (0.55)	1.33 (0.49)	1.09 (0.33)	−	−	−	−
vCOP ML (cm/s)		Unperturbed	0.65 (0.27)	0.87 (0.50)	0.88 (0.58)	0.63 (0.22)	−	−	−	−
		Obstacle	0.69 (0.30)	0.96 (0.55)	0.90 (0.53)	0.77 (0.24)	−	−	−	−
		Step−up	0.77 (0.30)	0.99 (0.40)	0.89 (0.48)	0.76 (0.29)	−	−	−	−
		Step−down	0.81 (0.34)	1.19 (0.66)	1.78 (1.06)	0,75 (0.34)	−	−	−	−
S1 (s)	Phase 2	Unperturbed	1.01 (0.30)	0.93 (0.21)	1.65 (0.20)	0,90 (0.17)	−	**+**	**+**	−
		Obstacle	1.04 (0.40)	1.04 (0.24)	1.81 (0.30)	1.01 (0.21)	−	**+**	**+**	−
		Step−up	1.02 (0.25)	0.91 (0.19)	1.62 (0.24)	0.92 (0.21)	−	**+**	**+**	−
		Step−down	0.99 (0.35)	0.89 (0.43)	1.84 (0,47)	1.21 (0.53)	−	**+**	**+**	−
S2 (s)		Unperturbed	0.91 (0.67)	0.85 (0.26)	1.72 (0.45)	0,90 (0.27)	−	**+**	**+**	−
		Obstacle	0.82 (0.66)	0.96 (0.39)	1.82 (0.52)	1.05 (0.48)	−	**+**	**+**	−
		Step−up	0.84 (0.66)	0.98 (0.50)	1.86 (0.92)	1.04 (0.41)	−	**+**	**+**	−
		Step−down	1.06 (0.63)	0.83 (0.39)	1.89 (0.65)	1.12 (0.45)	−	**+**	**+**	−
Transit time (s)		Unperturbed	2.28 (0.91)	2.08 (0.41)	3.79 (0.44)	2.15 (0.32)	−	**+**	**+**	−
		Obstacle	2.17 (1.01)	2.27 (0.55)	3.96 (0.70)	2.35 (0.67)	−	**+**	**+**	−
		Step−up	2.30 (0.89)	2.23 (0.67)	4.15 (1.18)	2.34 (0.57)	−	**+**	**+**	−
		Step−down	2.35 (0.91)	1.94 (0.72)	4.00 (0.84)	2.56 (0.94)	−	**+**	**+**	−
vCOP AP (cm/s)	Phase 3	Unperturbed	0.91 (0.28)	0.94 (0.25)	1.45 (0.54)	1,01 (0.21)	−	**+**	**+**	−
		Obstacle	1.01 (0.29)	0.93 (0.18)	1.68 (0.61)	1.01 (0.29)	−	**+**	**+**	−
		Step−up	1.17 (0.45)	1.12 (0,44)	1.69 (0.40)	1.07 (0.39)	−	**+**	**+**	−
		Step−down	1.09 (0.42)	1.1 (0.2)	1.60 (0.48)	1.19 (0.47)	−	**+**	**+**	−
vCOP ML (cm/s)		Unperturbed	0.84 (0.33)	0.94 (0.46)	1.38 (0.85)	0.86 (0.43)	−	−	**+**	−
		Obstacle	0.83 (0.37)	0.95 (0.32)	1.60 (0.92)	0.82 (0.38)	−	**+**	**+**	−
		Step−up	1.13 (0.68)	1.08 (0.48)	1.90 (1.24)	0.82 (0.38)	−	**+**	**+**	−
		Step−down	1.00 (0.50)	1.1 (0.5)	1.78 (1.06)	0.94 (0.47)	−	−	**+**	−

*COP, center of foot pressure; vCOP, velocity of COP; ap, antero-posterior direction; ml, medio-lateral direction; PD, Parkinson's disease; PD-II, participants with mild PD; CA, control group A; PD-III, participants with moderate PD; CB, control group B; SD, standard deviation, significant differences are reported in **“+”** (p <0.05). No significant differences are reported in – (p > 0.05)*.

### Impact of Testing Conditions on Trial Performance

**Figure 4:**
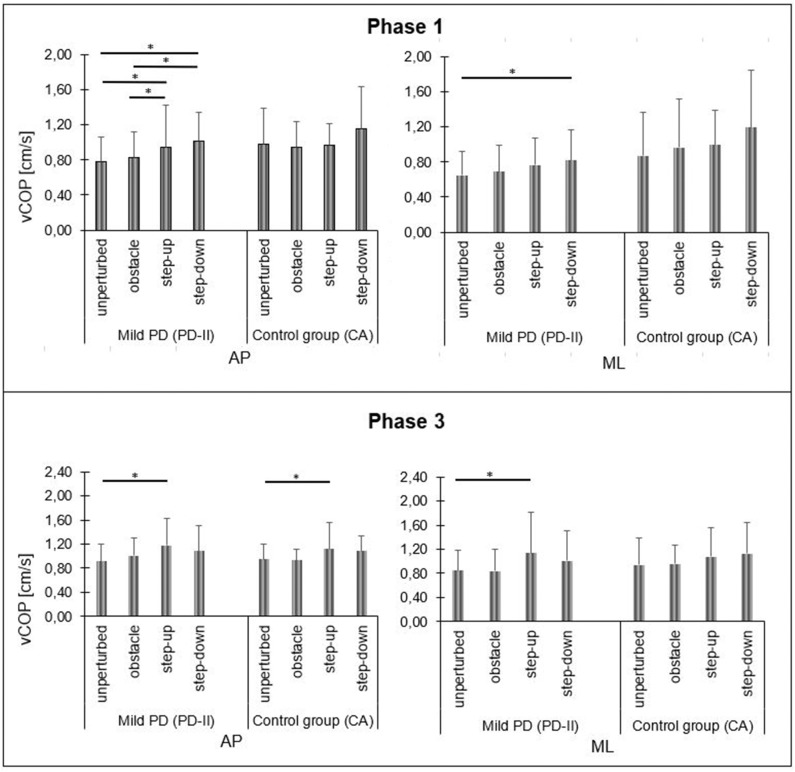
Mild PD and control group A vCOP changes in sagittal (AP) and frontal (ML) planes during quiet standing before (phase 1) and after (phase 3) the transitional phase, depending on test conditions. Significant differences are reported in *p < 0.05.

In mild PD participants, the repeated measures one-way ANOVA test revealed the significant impact of the testing conditions on phase 1 antero-posterior COP velocity [F_(3, 42)_ = 6.98; *p* < 0.001]. The phase 1 COP velocity for the sagittal plane was significantly higher in the step-up and step-down condition compared to the unperturbed condition and obstacle crossing trial. Also, there was a significant impact of the testing conditions on phase 1 medio-lateral COP velocity [F_(3, 42)_ = 3.22; *p* = 0.03]. The phase 1 COP velocity for the frontal plane was significantly higher in the step-down condition compared to the unperturbed condition. The repeated measures one-way ANOVA test did not reveal any significant impact of the testing conditions on transit time, S1, and S2 (*p* > 0.05). In phase 3, statistical analysis revealed the significant impact of testing conditions on COP velocity for the sagittal plane [F_(3, 42)_ = 3.51; *p* = 0.02]. The phase 3 COP velocity for the sagittal plane was higher in the step-up trial compared to the unperturbed transit. There was a significant impact of the testing conditions on the phase 3 COP velocity for the medio-lateral direction [F_(3, 42)_ = 2.88; *p* = 0.05]. The phase 3 COP velocity for the frontal plane was higher in the step-up trial compared to the unperturbed transit (Figure 4).

In healthy subjects (CA), statistical analysis did not reveal any significant differences in phase 1 COP velocity for the sagittal plane between the testing conditions [F_(3, 42)_ = 1.56; *p* = 0.21]. Also, there was no significant impact of the testing conditions on the phase 1 COP velocity for the frontal plane [F_(3, 42)_ = 2.44; *p* = 0.08]. There was no significant impact of the testing conditions on transit time, S1, and S2 (*p* > 0.05). In phase 3, statistical analysis revealed the significant impact of the testing conditions on COP velocity for the sagittal plane [F_(3, 42)_ = 3.35; *p* = 0.03]. The phase 3 COP velocity for the sagittal plane was significantly higher in the step-up trial compared to the unperturbed transit. The repeated measures one-way ANOVA test did not reveal any significant impact of the testing conditions on phase 3 COP velocity for the frontal plane [F_(3, 42)_ = 2.60; *p* = 0.07] (Figure 4).

### Participants With Moderate Parkinson's Disease

The phase 1 COP velocity in the antero-posterior and medio-lateral directions for all conditions did not differ significantly between the groups (*p* > 0.05). In moderate PD participants (PD-III), the transitional phase variables (transit time, S1, S2) were significantly higher compared to people without neurological deficits (CB) for all testing conditions (*p* < 0.05). The phase 3 COP velocity in both the antero-posterior and medio-lateral directions was significantly higher in the moderate PD individuals compared to the control group (CB) in crossing obstacle and step-up conditions (*p* < 0.05) (Table 2).

### Impact of Testing Conditions on Trial Performance

**Figure 5:**
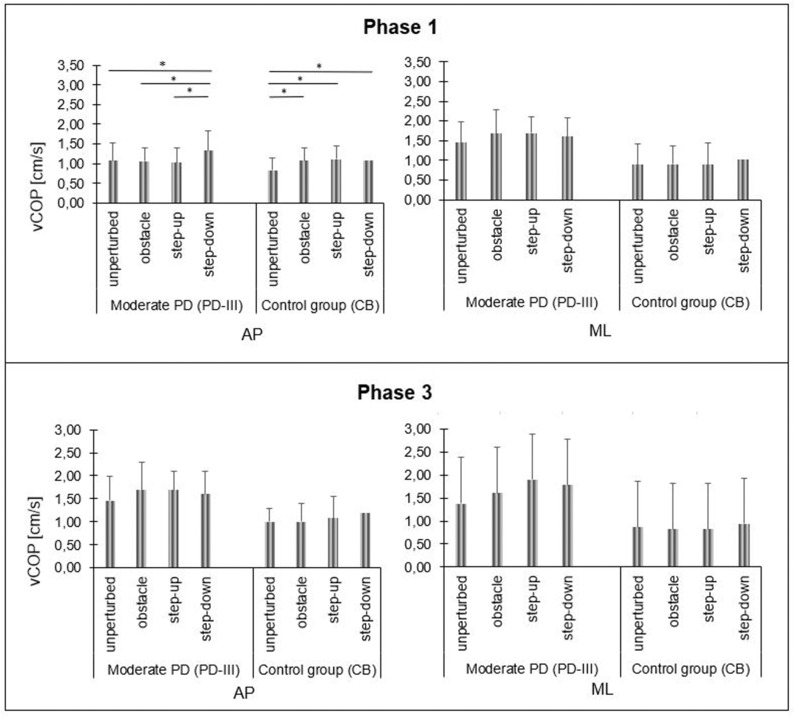
Moderate PD and control group B vCOP changes in sagittal (AP) and frontal (ML) planes during quiet standing before (phase 1) and after (phase 3) the transitional phase, depending on test conditions. Significant differences are reported in *p < 0.05.

**Figure 6:**
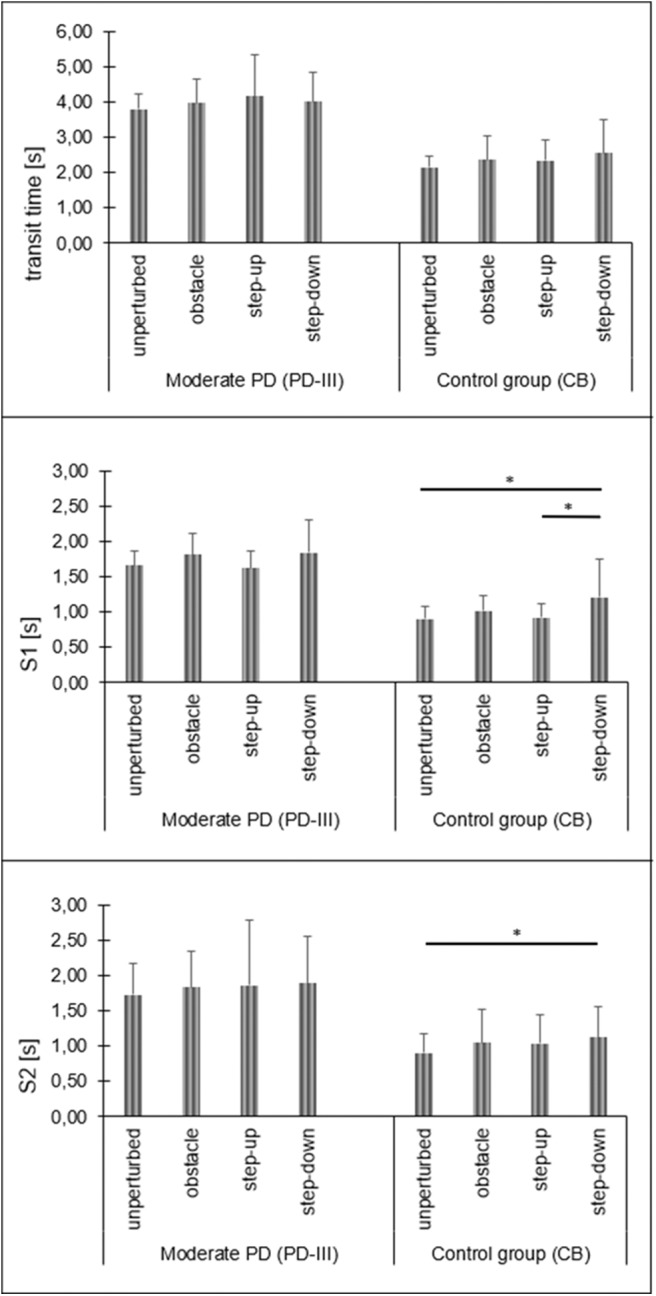
Moderate PD and control group transit time, stability time 1 and stability time 2 changes depending on test conditions (phase 2). Significant differences are reported in *p < 0.05.

In moderate PD participants, the repeated measures one-way ANOVA test revealed the significant impact of the testing conditions on phase 1 COP velocity for the sagittal plane [F_(3, 42)_ = 9.61; *p* < 0.001]. The step-down phase 1 COP velocity for the sagittal plane was significantly higher compared to all testing conditions. There was no significant impact of the testing conditions on phase 1 COP velocity for the frontal plane [F_(3, 42)_ = 1.19; *p* = 0.33]. In moderate PD participants, statistical analysis did not reveal any significant impact of the testing conditions on all study variables during phase 2 and phase 3 (*p* > 0.05) (Figures 5, 6).

In healthy subjects (CB), the repeated measures one-way ANOVA test revealed the significant impact of the testing conditions on phase 1 COP velocity for the sagittal plane [F_(3, 42)_ = 8.62; *p* < 0.001]. The phase 1 COP velocity for the sagittal plane during quiet standing in the obstacle crossing, step-up, and step-down trials was significantly higher compared to the unperturbed transit. There was no significant impact of the testing conditions on phase 1 COP velocity for the frontal plane [F_(3, 42)_ = 2.65; *p* = 0.06]. Statistical analysis revealed the significant impact of the testing conditions on S1 [F_(3, 42)_ = 3.84; *p* = 0.02] and S2 [F_(3, 42)_ = 3.01; *p* = 0.04]. The S1 of the step-down trial was significantly longer compared to the unperturbed transit and step-up trials. The S2 of the step-down trial was significantly longer compared to the unperturbed transit. In phase 3, statistical analysis did not reveal any significant impacts of the testing condition on COP velocity for both the sagittal [F_(3, 42)_ = 2.82; *p* > 0.05] and frontal [F_(3, 42)_ = 2.39; *p* = 0.08] planes (Figures 5, 6).

### Comparison of Participants With Mild and Moderate Parkinson's Disease

The phase 1 COP velocity in the antero-posterior direction did not differ significantly between moderate PD individuals and mild PD participants for obstacle, step-up and step-down conditions (*p* > 0.05). However, phase 1 COP velocity for sagittal plane was significantly higher in moderate PD relative to mild PD in flat condition (*p* = 0.003). In the moderate PD group, the transitional phase variables (transit time, S1, S2) were significantly higher compared to mild PD individuals for all testing conditions (*p* > 0.05). The phase 3 COP velocity in both the antero-posterior and medio-lateral direction was significantly higher in moderate PD participants compared to mild PD for the unperturbed condition, obstacle crossing, and the step-up and step-down conditions (*p* < 0.001) (Table 2).

## Discussion

Individuals with PD present an elevated risk of falls during activities associated with daily living, such as walking, turning, standing up from sitting, crossing obstacles, and during step initiation (5–7, 9–11, 21). In our study, we investigated a transitional task under different conditions in people with mild and moderate PD. We found that individuals with moderate PD experience difficulties in the transitional task during daily activities. In addition, we have observed that mild PD individuals did not show impairments during the transitional phase in the presence of different constraints. However, our study found that, independent of disease stage, the parameters of transitional tasks change with the increasing difficulty of these tasks. The most demanding tasks for all groups were ascending and descending steps.

The main findings in our study comprised no significant difference between mild PD individuals and healthy older adults in COP velocity for both the antero-posterior and medio-lateral directions during quiet standing before the transitional task and after completing the task. The quantification of balance disorders in PD is a challenge, especially for identifying abnormalities in the early stages of PD. Some authors have suggested that posturography can be a useful measure of postural instability (24, 42). There is evidence that balance disorders exist in people with PD in H&Y stages I-II; Beuter et al. (25) revealed that the early stages of PD affect body sway parameters, and similarly Chastan et al. (43) found that mild PD affects postural sway in the medio-lateral direction. Both of the mentioned studies have shown that postural control is affected early in the disease progression, however, in our case we have noticed only a tendency toward lower COP velocity for both the antero-posterior and medio-lateral directions in mild PD participants. In addition, the regained stability time after the transitional phase and the postural preparation time were comparable to healthy adults. Similar to our results, Carpinella et al. (44) and Mancini et al. (30) showed similar anticipatory postural adjustment durations for PD and control subjects. However, both studies found a significantly smaller lateral and backward COP displacement during the anticipatory postural adjustment in subjects with untreated (30) and early PD (44) compared to control subjects. The authors suggested that Parkinson's disease might affect the loading/unloading of the legs early in the disease. In addition, there is clear evidence that impairments are also present during other transitional tasks such as turning (44).

Nonetheless, we noticed that in mild PD participants, there was a significant impact of the testing conditions on COP velocity in the sagittal plane. Before the transitional phase in the step-up and step-down tasks, the early-stage PD participants demonstrated higher postural sway compared to the unperturbed condition and obstacle crossing, which may indicate that mild PD individuals present difficulties during stairs negotiation. In the recent literature there is evidence that PD individuals are at higher risk of falls when descending stairs (7) because of a reduced ability to produce adequate muscle strength, in particular reduced strength for the knee extensors (6). Moreover, a recent study investigated transitional tasks from quiet standing to step climbing (21, 45). The authors noticed reduced step frequency and a significant reduction of the medio-lateral acceleration in PD individuals undergoing a step climbing task compared to level ground walking. In our case, we also noticed postural changes as the COP velocity increased after the ascending step in respect to the unperturbed transitional task. Walking on stairs is a common daily task that requires complex motor control, including postural control and movement coordination. It is important to investigate this aspect, especially in PD, because it is well known that PD individuals present problems with balance, coordination, and programming movement (27, 46). Nevertheless, there were no differences between the unperturbed condition and obstacle crossing, which may indicate that mild PD participants do not present difficulties with crossing obstacles. This is confirmed by research from Vitorio et al. (37), where the effect of PD severity on crossing an obstacle was examined. The authors showed that people experiencing a mild stage of PD did not present balance disorders while negotiating an obstacle.

Another major finding in this study was that the moderate PD individuals presented higher COP velocity in both the sagittal and frontal planes after making a step compared to healthy older people. Furthermore, the moderate PD group showed higher values of the transitional phase variables. There is convincing evidence that the incidence of postural instability and difficulty of gait initiation increase with PD severity (1, 20, 31). It is known that in moderate PD, step initiation is associated with bradykinesia and prolonged preparatory COP displacements (47). Our study supports these findings; the PD participants present larger values of transit time and preparatory postural time (S1), which may indicate bradykinesia. This is in agreement with previous reports (47, 48) where PD individuals demonstrate prolonged preparation time and slower stride velocity with respect to healthy older people. These abnormalities in gait initiation may reflect deficits in selected aspects of motor programming as well as motor planning (47). It has been well investigated that the basal ganglia pathways support the preparation of simple movements; however, they seem to play a particular role in the preparation and performance of sequential movements (49), which contain the step tasks under different conditions. Our results indicate problems with the transitional task and confirmed that the basal ganglia pathways are dysfunctional in PD and that their role in the motor control of voluntary movements is impaired (50). Additionally, our study revealed that people with moderate PD have difficulties with regaining stability after transitional phase. We observed that PD participants presented greater values of stability time 2 and higher sway velocity after completing the transitional task compared to the control group. This may be related to actively braking a step, and thus the active control of antigravity muscles that occur before foot contact of the swinging limb (49), which is impaired in PD individuals (51). Moreover, Chastan et al. (51) analyzed step length and anteroposterior and vertical velocities of the center of gravity during gait initiation. The authors recorded that during the swing limb period, healthy subjects showed a fall in the center of gravity which was then reversed before foot contact, indicating active braking. In PD participants, the step length and velocity were significantly reduced and no braking occurred before foot contact. In our case, we analyzed different postural measurements than these authors; however, from our results, we may assume that increased postural sway and a longer time to achieve a stable posture after the transitional phase are related to impaired postural and antigravity muscle control.

Our results demonstrate the effect of different constraints on COP velocity for both the sagittal and frontal planes before the transitional phase. Moderate PD participants presented higher sway velocity before the step-down transition compared to the unperturbed condition, obstacle crossing, and step-up trial. It is established that people with PD report less confidence in their ability to perform activities of daily living (ADLs) without falling (12), so our results suggest that descending stairs is the most demanding task for PD individuals, which may be associated with a fear of falling. Additionally, several studies have reported that people with PD adopt different strategies to cross obstacles compared to healthy older people (23, 35, 52). Some people with PD walk with greater and faster medio-lateral sway than age-matched older individuals (23). Stegemoller et al. (35) reported disease-dependent decreases in velocity, step length, and antero-posterior range of motion of the center of mass (COM) in individuals with PD when stepping over an obstacle. Our results confirmed the findings of these authors; moreover, we noticed that all testing conditions have an impact on preparatory postural time, stride time, and stability regain time in moderate PD subjects, who demonstrate similar values of the abovementioned parameters in the obstacle crossing, step-up, and step-down condition tasks. Additionally, compared to mild PD participants and healthy older people, people with moderate PD presented a longer preparation time and stride time and exhibited difficulties with regaining stability after a step during all disruption tasks. Our results confirmed previous studies that advanced PD individuals demonstrate balance disorders during gait with an additional task (22, 53, 54). As expected, unperturbed transit between platforms was the simplest task for all groups.

In summary, the PD-III group showed higher values of posturographic parameters during the transitional task. Disease severity affects postural control during dynamic activities such as initiating gait in PD. Our study revealed characteristic abnormalities in the transitional which that may reflect bradykinesia and general hypokinesia in PD. People with moderate PD demonstrate deficits in some aspects of motor planning and initiating and executing movement. Likewise, PD participants present difficulties with regaining stability after making a step during different disruptions, which may manifest the loss of postural reflexes.

Although the results of this study are consistent and essential, this study has certain limitations. The number of participants was small, especially regarding individuals with PD in the early stage of disease (H&Y stage I). We suggest that a larger number of subjects at this stage of PD should be investigated to confirm our findings. Second, individuals with Parkinson's disease were tested during the “ON period,” having taken their usual antiparkinsonian medication, so the impact of pharmacological treatment on the characteristics of postural control was not taken into account. Therefore, in the future, a study should be carried out during both the ON and OFF periods, which would provide additional information on the characteristics of postural control in people with PD. Additionally, the freezing of gait (FoG) may affect gait initiation, which was not investigated in our study. During step initiation, freezers use a more restrictive postural strategy and are slower compared to both healthy subjects and non-freezers. Freezers are also slower to initiate gait during conflicting conditions compared to nonconflicting conditions, and they implement inappropriate motor programs, both during gait initiation and gait inhibition (55). Therefore, in future research, it is necessary to use our procedure in freezer and non-freezer groups. Nevertheless, the importance of our results is not diminished since the selection of subjects and postural evaluations were performed according to standard and reliable scientific procedures.

## Conclusions

The procedure of performing a transitional task under different conditions allowed us to detect differences among mild and moderate PD stages and people without neurological deficits. Posturography is an effective tool for detecting abnormalities in a transitional task; moreover, it is an efficient method for differentiating the severity of Parkinson's disease.

The results suggest that the proposed procedure is a promising tool for evaluating selected aspects of a transitional task, allowing the assessment of balance disorders not only in scientific research but also in the clinical evaluation. Therefore, it is possible to use the described method in medical practice without using more advanced equipment such as an optoelectronics system, which is used mostly in research.

## Data Availability Statement

The datasets generated for this study are available on request to the corresponding author.

## Ethics Statement

The studies involving human participants were reviewed and approved by The ethics committee of the Jerzy Kukuczka Academy of Physical Education in Katowice, Poland (No.7/2013/26.06.2013). The patients/participants provided their written informed consent to participate in this study.

## Author Contributions

GJ, AK-K, and MR-B designed the study. AK, JM, and AA performed the experiments. AK, WM, and JM collected data and analyzed data. AK, KS, and GJ wrote and edited manuscript.

## Conflict of Interest

The authors declare that the research was conducted in the absence of any commercial or financial relationships that could be construed as a potential conflict of interest.
